# The psychological mechanisms of the better-than-average effect in the moral and competence domains under self-enhancement and self-protection motives among young Japanese adults

**DOI:** 10.3389/fpsyg.2024.1367568

**Published:** 2024-10-22

**Authors:** Yi Ding, Motoaki Sugiura

**Affiliations:** ^1^Institute of Development, Aging and Cancer, Tohoku University, Sendai, Japan; ^2^Graduate School of Medicine, Tohoku University, Sendai, Japan; ^3^Japan Society for the Promotion of Science, Tokyo, Japan; ^4^International Research Institute of Disaster Science, Tohoku University, Sendai, Japan

**Keywords:** better-than-average effect, motivation, social perception dimension, personality trait, social acceptance, mutual trust

## Abstract

The better-than-average effect (BTAE) refers to the phenomenon where individuals perceive themselves as better than the average person. This effect has been independently examined in terms of social perception dimension and motivation. Additionally, no psychobehavioral traits have been found to be associated with the BTAE in the moral domain. However, the interactive effects of social perception dimension and motivation on the BTAE remain unclear, and its association with a broad range of psychobehavioral traits has not been extensively validated. In this study, we assess self-and average other-evaluations across four domains, based on two social perception dimensions and two motivations, to investigate their interactive effects on the BTAE (*n* = 678). We measured seven sets of psychobehavioral characteristics to examine their association with the BTAE, as well as self-and other-evaluations. Results indicated that the BTAE occurred only under negative moral conditions, while the worse-than-others effect was observed under two competence conditions. Furthermore, the BTAE was associated with only a few psychobehavioral characteristics in the moral domains, compared to many in the competence domains. Notably, both self-and other-evaluations were correlated with many trust-relevant characteristics in the moral domains. These findings suggest that sociocultural dynamics may influence the BTAE differently across various domains.

## Introduction

Most people view themselves through “rose-tinted glasses,” believing they are superior to the average person. This phenomenon is known as the better-than-average effect (Alicke et al., 1995). For example, people generally perceive themselves as more talented, moral, lucky, and healthy than their peers. Previous studies have examined the influence of different social perception dimensions (i.e., morality and competence) and motivations (self-enhancement and self-protection) on the better-than-average effect (BTAE) (Tappin and McKay, 2017; Zell et al., 2020). They found that the BTAE was stronger in the context of morality compared to competence and more pronounced under self-enhancement motive as opposed to self-protection motive. However, these studies have not explored the interactive effects of social perception dimension and motivation on the BTAE.

Moreover, personality psychology researchers have examined the correlation between BTAE and personality traits, leading to two contradictory perspectives. One perspective suggests that BTAE in the moral domain is a uniquely prevalent illusion with no significant correlation to personality traits (Tappin and McKay, 2017). In contrast, another perspective proposes the “self-centrality breeds self-enhancement” principle, which asserts that BTAE is correlated with personality traits that an individual perceives as important in that domain (Campbell et al., 2002). However, both lines of research have used only a limited number of personality traits within each domain and employed different measurement methods.

### Literature review

BTAE was first described as a population-level phenomenon in 1985 (Alicke, 1985) and has since been repeatedly confirmed (Ziano et al., 2020). The BTAE is robust and pervasive across age groups (Kurman, 2002; Albery and Messer, 2005; Zell and Alicke, 2011; Zell et al., 2020), occupations (Van Lange et al., 1997; Bergquist, 2020; Linder and Sperber, 2020), and cultures (Sedikides et al., 2003; Cai et al., 2011). Even prisoners have been found to believe they are better than the average prisoner and average community members on prosocial traits (Sedikides et al., 2014). Recently, many studies have investigated the BTAE in the context of perceptions of the economy, coping behavior, and other people during COVID-19 (Barrafrem et al., 2020; Hoorens et al., 2022; Kim and Han, 2023).

### Domains of BTAE: social perception dimension and motivation

The BTAE has been examined in the context of different social perception dimensions and motivations to understand the underlying psychological mechanisms. Morality and competence are two fundamental dimensions of the BTAE. Moral traits (e.g., kindness and cruelty) affect others directly, whereas competence traits (e.g., intelligence and stupidity) primarily benefit the individual possessing them (Wojciszke, 2005). A previous study has found a stronger BTAE in the domain of morality compared to competence when evaluating personality trait adjectives (Tappin and McKay, 2017). Considering the inherent value of morality, individuals tend to prioritize their moral virtues over their abilities, as moral traits have a direct impact on others and are more highly valued in social interactions than competence traits (Abele et al., 2021).

Self-enhancement and self-protection motives are the two main drivers of the BTAE (Sedikides et al., 2015). Self-enhancement motive leads individuals to improve their self-views (e.g., “I am more virtuous than others”), while self-protection motive prompts individuals to defend themselves and avoid negative self-views (e.g., “I am less immoral than others”). Previous studies have suggested that the strength of the BTAE is greater for positive traits compared to negative traits, indicating that the self-enhancement motive is more prominent than the self-protection motive (Zell et al., 2020). The differing effects of these motives suggest a behavioral preference for emphasizing positive traits rather than rejecting negative ones.

In summary, social perception dimension and motivation are two key factors influencing the BTAE. The social perception dimension pertains to how individuals perceive their own value within society, while motivation drives behaviors aimed at maintaining a positive self-concept.

### Relationships between BTAE and psychobehavioral characteristics

There are currently two prevailing perspectives on the correlation between BTAE and psychobehavioral characteristics. The first perspective, known as the “self-centrality breeds self-enhancement” principle, posits that there is a correlation between the BTAE and personality traits that an individual perceives as important in that domain (Campbell et al., 2002; Gebauer et al., 2013; Sedikides and Alicke, 2018). For example, narcissism and self-esteem are positively associated with the BTAE in the competence domain, while only self-esteem is related to the BTAE in the moral domain (Campbell et al., 2002). This perspective suggests that narcissists, who focus on competence and are less concerned with moral issues, do not rate themselves as more moral than others. The second perspective emphasizes the lack of associations between the BTAE and personality traits in the moral domain, proposing that the BTAE is a “uniquely prevalent illusion” in this domain (Tappin and McKay, 2017). No associations were found between the BTAE and two characteristics (i.e., self-esteem and moral identity) in the moral domain. This perspective argues that the BTAE in the moral domain is unique (i.e., independent of self-perceived personality) and prevalent (i.e., observed across people), and may be explained by an illusory psychological process that is detached from actual self-perception.

### Unsolved issues

Although the BTAE has been studied in the context of various social perception dimensions to clarify the underlying psychological mechanisms, several important issues remain. First, while previous studies have compared the BTAE across different social perception dimensions or motivations, the interactive effects of these dimensions on the BTAE have not been examined. People may be motivated by specific motives within particular social perception dimensions. For example, although meta-analyses have shown that the self-enhancement effect is generally stronger than the self-protection motive (Zell et al., 2020), individuals are often more inclined to refrain from immoral behavior rather than engage in moral behavior. This suggests that the self-protection motive may predominate in the moral domain (Klein and Epley, 2016).

Second, the contradictory findings regarding the association between BTAE in the moral domain and personality traits may be due to the limited number of personality traits studied. The debate surrounding the BTAE–personality relationship focuses on whether the BTAE in the moral domain is independent of self-perceived moral personality traits. Researchers have primarily examined limited traits, such as self-esteem and moral identity, as central traits in the moral domain (Campbell et al., 2002; Tappin and McKay, 2017). We suspect that their failure to find a significant link may be because these traits do not adequately capture the centrality of self in the moral domain. To better understand the uniquely prevalent illusion of the BTAE, it is essential to explore it in relation to a broader range of personality traits.

Third, differences in BTAE measurement methods should be considered in the context of the BTAE–personality relationship. The studies reporting strong associations between the BTAE and moral personality traits used direct measures, asking participants to assess the BTAE directly (Campbell et al., 2002). In contrast, the study reporting an independent relationship used an indirect method, where participants assessed themselves and others separately to calculate the BTAE (Tappin and McKay, 2017). Since self-and other-evaluations are known to be influenced by personality traits (Wood et al., 2010), the BTAE–personality association might be obscured in indirect measures. This issue may not arise with direct measures, where participants primarily focus on their self-evaluation (Klar and Giladi, 1999).

### Purpose

The present study aimed to investigate the psychological mechanisms underlying the BTAE by addressing the three issues described above. We conducted an online survey to assess self-and other-evaluations under positive/negative moral conditions and positive/negative competence conditions, as well as to evaluate seven sets of psychobehavioral characteristics.

First, we investigated the interactive effect of social perception dimension and motivation on the BTAE. We examined the presence of BTAE across four domains formed by combining two social perception dimensions and two motivations. We hypothesized that the BTAE would have a stronger effect under the negative moral condition compared to the positive moral condition, as suggested by a moral judgment study (Klein and Epley, 2016), whereas its effect might differ in the competence domain.

Second, we examined 22 psychobehavioral characteristics related to morality and competence to clarify the contradictory findings regarding the association between BTAE in the moral domain and psychobehavioral characteristics. In addition to self-esteem, moral identity, and narcissism scales used in previous studies, we included four additional scales. Based on a previous study (Tappin and McKay, 2017), we anticipated no significant associations between psychobehavioral characteristics and the BTAE in the moral domain, while expecting significant correlations in the competence domain.

Third, we explored the correlations between self-and other-evaluations and psychobehavioral characteristics to investigate the potential reasons for the “uniquely prevalent illusion” resulting from different BTAE measurement methods. We anticipated finding similar correlations between self-and other-evaluations in the moral domain and psychobehavioral characteristics.

## Materials and methods

### Participants

We conducted an online survey using the services of NEO Marketing (Tokyo, Japan). To achieve a power > 90% (*d* = 0.2, *α* = 0.05) and address the potential limitations of online survey data (Chmielewski and Kucker, 2020), we needed to recruit 1,300 participants; ultimately, we recruited 1,272. We first excluded eight participants whose responses were inconsistent between registered and reported demographic information, 141 satisfiers (i.e., people who only met the minimum requirement to finish the survey or who responded thoughtlessly) who provided the same responses to all questions, 123 satisfiers who responded “not at all seriously” to the question “*How seriously are you answering the questionnaire?*,” and 322 satisfiers whose reaction times were less than 8 min (based on the internal consistency of three well-established scales; see Supplementary material for further details regarding this criterion, and for the results of analyses not excluding satisfiers). Thus, valid data from 678 participants were analyzed (307 males; age range: 20–29 years; mean age = 25.79 ± 2.79 years). We report all measures, manipulations, and exclusions in this study. The data, R code, analyses, and Supplementary material can be retrieved from the OSF platform (doi: 10.17605/OSF.IO/XM5HW).

### Materials

We conducted a preliminary survey to ensure the validity of the 80 domain-specific adjectives used as stimuli. One group of participants was instructed to categorize 592 personality trait adjectives from two corpora into four domains, and we finally obtained 160 adjectives (Aoki, 1971; Murakami, 2002). Another group of participants was asked to rate the 160 adjectives according to three properties (social perception dimension, valence, and familiarity). Ultimately, 80 adjectives were selected as stimuli. The details of this survey and the word list are provided in Supplementary material. Table 1 shows the mean social perception dimension, valence, and familiarity scores of the adjectives in the four domains.

**Table 1:** Average social perception dimension, valence, and familiarity scores in the four domains (standard deviation is in parentheses).

	Moral positive	Moral negative	Competent positive	Competent negative
Social perception dimension	2.414 (0.249)	2.748 (0.121)	5.287 (0.289)	4.629 (0.464)
Valence	5.811 (0.535)	1.843 (0.234)	5.549 (0.439)	2.411 (0.333)
Familiarity	5.378 (0.686)	4.739 (0.417)	5.509 (0.455)	5.091 (0.476)

Social perception dimension scores ranged from 1 (very moral) to 7 (very competent); valence scores ranged from 1 (very positive) to 7 (very negative), and familiarity scores ranged from 1 (not familiar at all) to 7 (very familiar).

### Measures

Participants completed an online consent form and were presented with questions about basic demographics (sex and age), self-and average other-evaluations (160 items), and psychobehavioral characteristics (148 items), as well as an attention test.

### Self-and average other-evaluations

**Figure 1:**
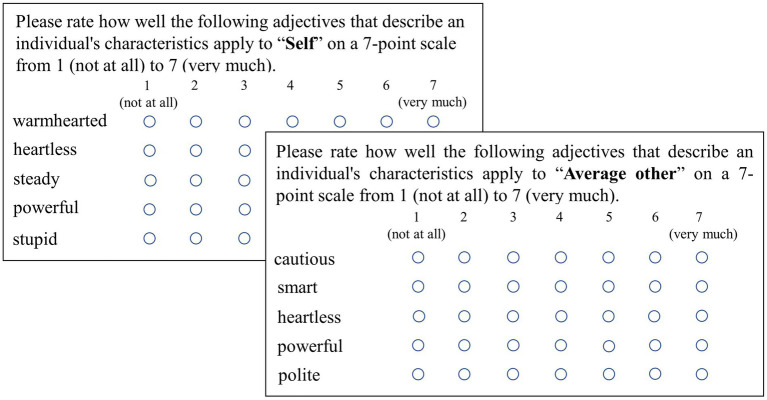
Self-evaluation and average other-evaluation instrument.

Participants were instructed to complete the self-and average other-evaluations (i.e., identity). Both evaluations used the same 80 adjectives counterbalanced by social perception dimension (i.e., morality or competence) and motivation (i.e., positive or negative). The order in which the adjectives were presented was randomized across participants for both evaluations. As shown in Figure 1, participants were instructed to rate the extent to which each adjective described them personally, or others of the same sex and age, from 1 (*not at all*) to 7 (*very much*).

### Psychobehavioral characteristics

The internal consistency of the scales was measured using Cronbach’s *α* (range: 0.65–0.93).

Self-esteem was measured using the 10-item Rosenberg Self-Esteem Scale (Rosenberg, 1965), which has been translated into Japanese (Yamamoto et al., 1982). Items included statements such as “I’m positive about myself” (Cronbach’s *α* = 0.89). Participants rated items from 1 (*not at all*) to 5 (*very much*).

Moral identity was measured using the nine-item Moral Identity Scale (MI; Aquino and Reed, 2002), which has been translated into Japanese (Kawamura et al., 2017). This scale quantifies the importance ascribed to morality concerning internalization (e.g., “becoming a person with these characteristics will make me feel better”; Cronbach’s *α* = 0.75) and symbolization (e.g., “by looking at the groups I belong to, people around me will think that I have these characteristics”; Cronbach’s *α* = 0.81). Participants rated items using a 5-point scale ranging from 1 (*I do not think so at all*) to 5 (*I very much think so*).

Narcissism was measured using the 35-item Narcissistic Personality Inventory (NPI; Raskin and Terry, 1988), which has been translated into Japanese (Konishi et al., 2006). This inventory assesses the need for attention (e.g., “I want to show myself in a positive light when I have a chance”; Cronbach’s *α* = 0.90); a sense of grandeur (e.g., “I’m a person with a special talent”; Cronbach’s *α* = 0.92); leadership (e.g., “I think I’m a good leader”; Cronbach’s *α* = 0.93); positive regard toward the body (e.g., “I like to see my body”; Cronbach’s α = 0.87), and self-conviction (e.g., “I always understand my behavior”; Cronbach’s α = 0.75). Participants rated items using a 6-point scale ranging from 1 (*not at all*) to 6 (*very much*).

The 34-item Power to Live Scale includes eight moral- and competence-related factors that are advantageous for survival (Sugiura et al., 2015), including leadership (e.g., “To resolve problems, I gather together everyone involved to discuss the matter”; Cronbach’s *α* = 0.85), problem-solving (e.g., “When I am fretting about what I should do, I compare several alternative actions”; Cronbach’s *α* = 0.76), altruism (e.g., “When I see someone having trouble, I have to help them”; Cronbach’s α = 0.83), stubbornness (e.g., “I am stubborn and always get my way”; Cronbach’s α = 0.70), etiquette (e.g., “I take the initiative in greeting family members and people living in the neighborhood”; Cronbach’s α = 0.65), emotional regulation (e.g., “During difficult times, I endeavor not to brood”; Cronbach’s *α* = 0.68), self-transcendence (e.g., “I am aware that I am alive and have a sense of responsibility in life”; Cronbach’s α = 0.70), and active well-being (e.g., “In everyday life, I have habits that are essential for relieving stress or giving me a change of pace”; Cronbach’s *α* = 0.67). Participants responded using a 6-point scale ranging from 1 (*not at all*) to 6 (*very much*). The BTAE can be discussed in an evolutionary context.

Self-efficacy was measured using the 16-item GSE (Sakano and Mitsuhiko, 1986). This scale measures an individual’s general beliefs about their ability to solve a wide range of problems based on motivation to act (e.g., “I’m confident whenever I do something”; Cronbach’s *α* = 0.79), anxiety about failure (e.g., “After finishing work, I always feel that I have failed”; Cronbach’s α = 0.77), and social competence (e.g., “I have a better memory than others”; Cronbach’s α = 0.69). Participants responded “*yes”* or “*no”* to all items. This questionnaire can be used to explore the association between BTAE and general self-perceived competence.

Social desirability was measured using the 24-item Balanced Inventory of Desirable Responding (BIDR) instrument (Paulhus and Reid, 1991), which has been translated into Japanese (Tani, 2008). This scale measures self-deception, which involves exaggerating one’s competence (e.g., “I am a completely rational person”; Cronbach’s α = 0.78), and impression management, which involves presenting oneself in a morally favorable light (e.g., “If necessary, I sometimes lie”; Cronbach’s α = 0.68). Participants rated the items from 1 (*not at all*) to 7 (*very much*).

Depression was measured using the 20-item Center for Epidemiologic Studies Depression Scale (Radloff, 1977), which has been translated into Japanese (Shima et al., 1985). This instrument measures symptoms of depression in the preceding 2 weeks (e.g., “I thought my life was a failure”; Cronbach’s α = 0.91). Participants rated the items from 1 (*<1 day*) to 4 (*5–7 days*). The score is negatively associated with the BTAE score (Tabachnik et al., 1983; Taylor and Brown, 1988).

#### Attention test

Participants were asked “How seriously are you answering the questionnaire?” and responded on a scale ranging from 1 (*not at all seriously*) to 6 (*very seriously*).

### Analysis

Analyses were performed using R software (version 4.1.1; R Core Team, 2019) and the tidyverse (Wickham et al., 2019), psych (Revelle, 2019), reshape2 (Wickham, 2007), lsr (Navarro, 2015), ggplot2 (Wickham, 2016), and gridExtra (Auguie, 2017) packages.

### The BTAE in the four domains

We performed repeated-measures analysis of variance (ANOVA) to examine the effects of social perception dimensions, motivation, and identity, with the paired *t*-test being used to investigate interaction effects. Since we were interested in the differences between self- and other-evaluations in the four domains, we focused on the interactions with identity and used the Bonferroni method to correct for multiple comparisons (n = 4).

### Correlation analysis between psychobehavioral characteristics and the BTAE

The BTAE score represents the extent to which a participant views themselves as better/worse than others. Thus, for the two negative conditions, scores were reverse-coded such that higher scores were more positive. The BTAE score was calculated according to the difference between the self-and average other-evaluation scores. We conducted correlation analyses to explore the relationships between psychobehavioral characteristics and the BTAE in the four domains. Considering the large sample size, we set |*r|* > 0.3 as the effect size threshold (Cohen, 1992).

### Correlations analysis between psychobehavioral characteristics and self-/other-evaluations

We also conducted correlation analyses to explore the relationships between the psychobehavioral characteristics and self-and other-evaluations in the four domains. Considering the large sample size, we set |*r|* > 0.3 as the effect size threshold (Cohen, 1992).

## Results

### The BTAE in the four domains

**Figure 2:**
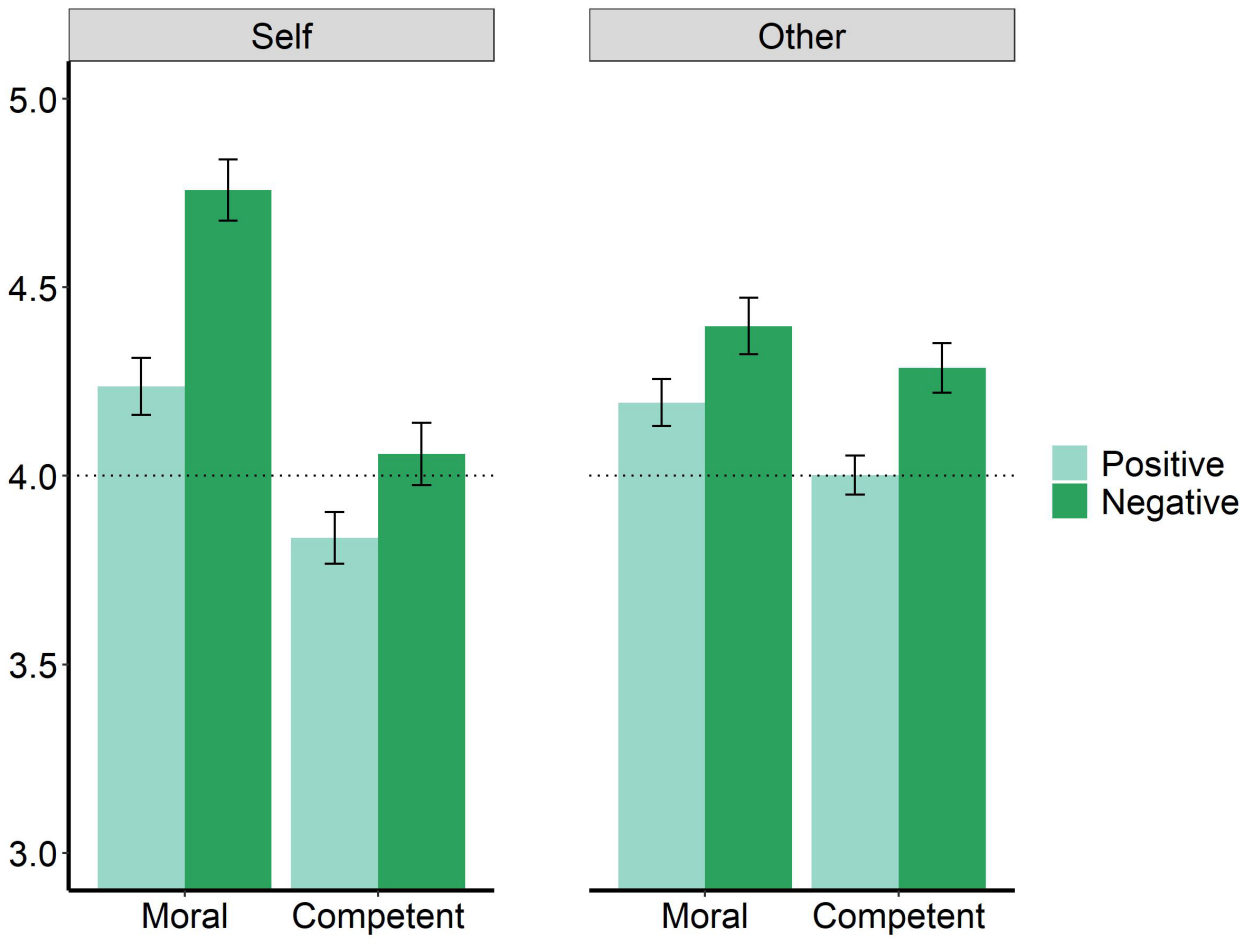
Self-evaluation and average other-evaluation scores under the four conditions. Error bars are the 95% CIs.

**Figure 3:**
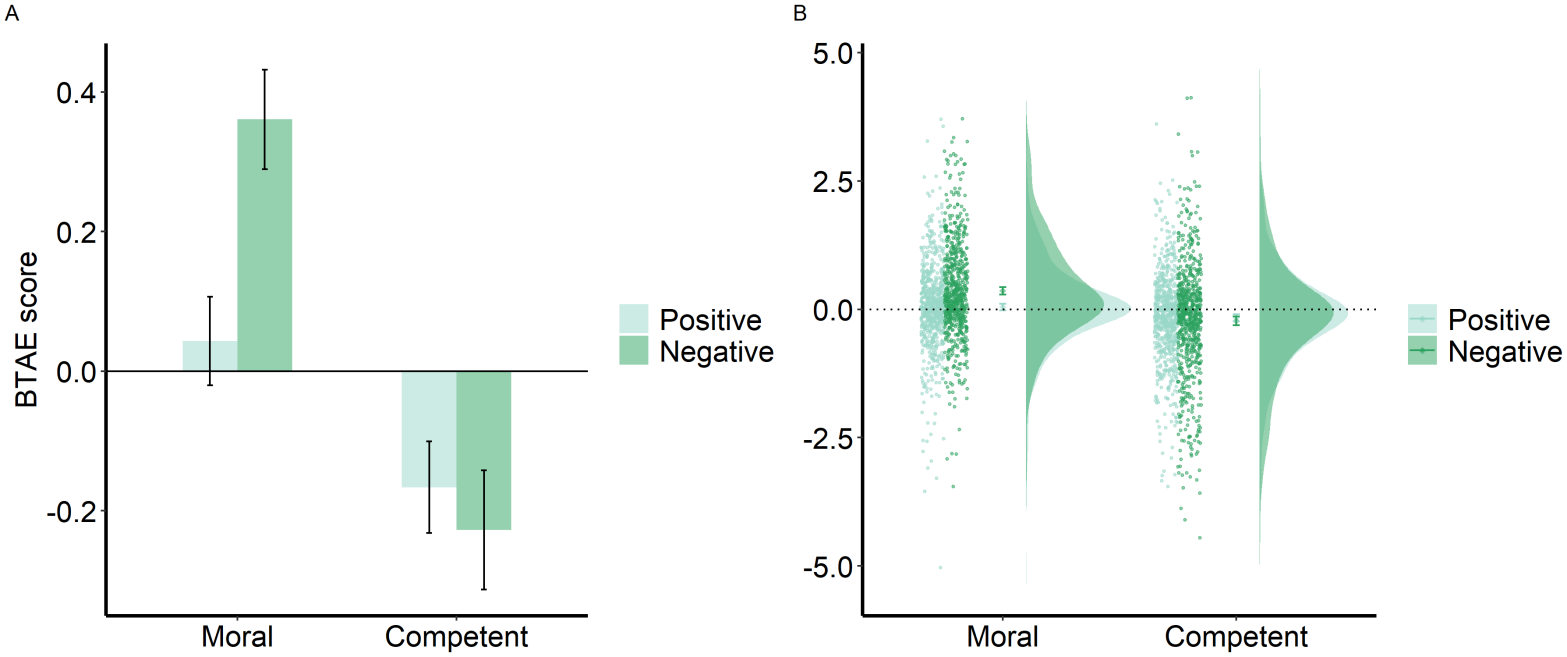
Raincloud plot. (A) The mean BTAE and (B) its distribution under the four conditions. Error bars are 95% CIs.

Figure 2 presents the mean self-evaluation and average other-evaluation scores. Figure 3 presents the mean BTAE in the four domains and the data points for each participant (Allen et al., 2021). The reliability coefficients for the four domains ranged from 0.92 to 0.96.

The results show that the BTAE occurred under the negative moral condition, and the worse-than-others effect (WTAE) occurred under the positive and negative competence conditions. The results of the repeated-measures ANOVA showed significant main effects of domain [*F*(1, 677) = 260.512, *p* < 0.001, η^2^_p_ = 0.278] and valence [*F*(1, 677) = 72.946, *p* < 0.001, η^2^_p_ = 0.097]. A significant interaction was detected between domain and identity [*F*(1, 677) = 132.653, *p* < 0.001, η^2^_p_ = 0.164]. A paired *t*-test showed that participants rated themselves as being better than others under the moral condition [*t*(677) = 6.879, *p* < 0.001, Cohen’s *d* = 0.264, 95% CI: 0.187, 0.341] but worse than others under the competence condition [*t*(677) = −5.864, *p* < 0.001, Cohen’s *d* = −0.225, 95% CI: −0.301, −0.149]. A significant interaction was observed between valence and identity [*F*(1, 677) = 15.672, *p* < 0.001, η^2^_p_ = 0.023], but no significant difference was found between self-and other-evaluations under the positive (*p* = 0.24) or negative (*p* = 0.376) condition. A significant three-way interaction was also detected [*F*(1, 677) = 100.910, *p* < 0.001, η^2^_p_ = 0.130]. Follow-up paired *t*-tests showed significant differences between self-and other-evaluations under the negative moral condition, where people rated themselves as less immoral than others [*t*(677) = 9.926, *p* < 0.001, Cohen’s *d* = 0.381, 95% CI: 0.303, 0.459], as well as under the positive and negative competence conditions, indicating that participants perceived themselves as less capable [*t*(677) = −4.956, *p* < 0.001, Cohen’s *d* = 0.190, 95% CI: 0.114, 0.266] and more incompetent than others [*t*(677) = −5.234, *p* < 0.001, Cohen’s *d* = 0.201, 95% CI: 0.125, 0.277]. The *p*-values were adjusted for multiple comparisons.

### Correlations between psychobehavioral characteristics and the BTAE

**Figure 4:**
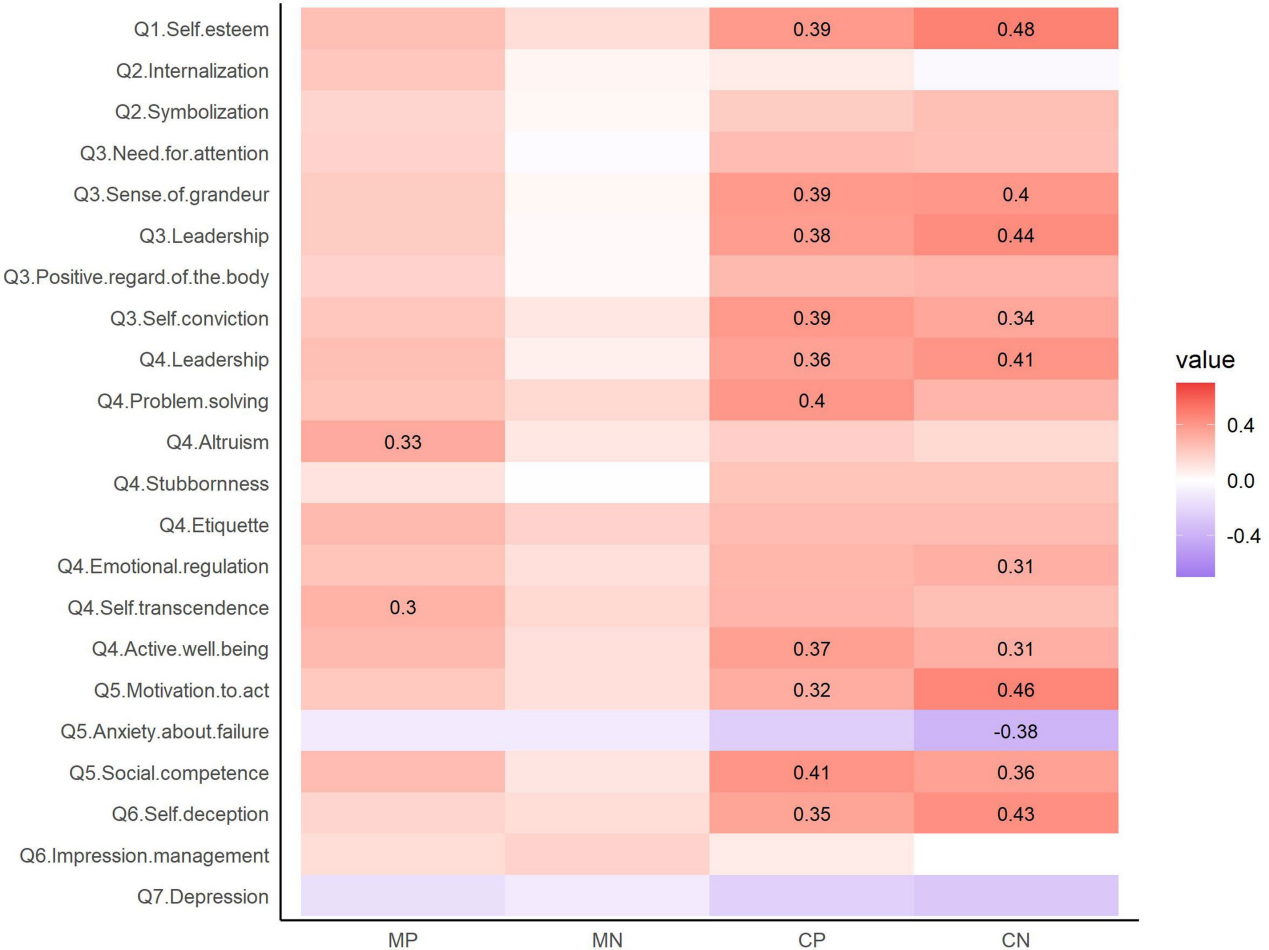
Heatmap of the correlation coefficients between the BTAE and the psychobehavioral characteristics. |*r*| > 0.3 are displayed. The color scale represents the correlation coefficient, with darker red indicating stronger positive correlations and darker blue indicating stronger negative correlations. MP, positive morality; MN, negative morality; CP, positive competence; CN, negative competence. Q1: Rosenberg Self-Esteem Scale, Q2: Moral Identity Scale, Q3: Narcissistic Personality Inventory, Q4: Power to Live scale, Q5: General Self-Efficacy Scale, Q6: Balanced Inventory of Desirable Responding Instrument, Q7: Center for Epidemiologic Studies Depression Scale.

The BTAE under the negative moral condition was not associated with any psychobehavioral characteristic, but the BTAE under the three other conditions was associated with some of the psychobehavioral characteristics (Figure 4). The BTAE under the positive moral condition was positively correlated with altruism and self-transcendence on the Power to Live Scale. However, none of the associations between BTAE under the negative moral condition and the psychobehavioral characteristics reached an |*r*| of 0.3 (the largest coefficient was for impression management on the BIDR; *r* = 0.172). The BTAE was associated with nine common characteristics under the positive and negative competence conditions, including self-esteem, sense of grandeur, leadership, and self-conviction on the NPI; leadership and active well-being on the Power to Live Scale; motivation to act and social competence on the GSE; and self-deception on the BIDR. The BTAE under the positive competence condition was also associated with problem-solving on the Power to Live Scale, and the BTAE under the negative competence condition was additionally associated with emotional regulation on the Power to Live Scale and anxiety about failure on the GSE.

### Correlations between psychobehavioral characteristics and self-/other-evaluations

**Figure 5:**
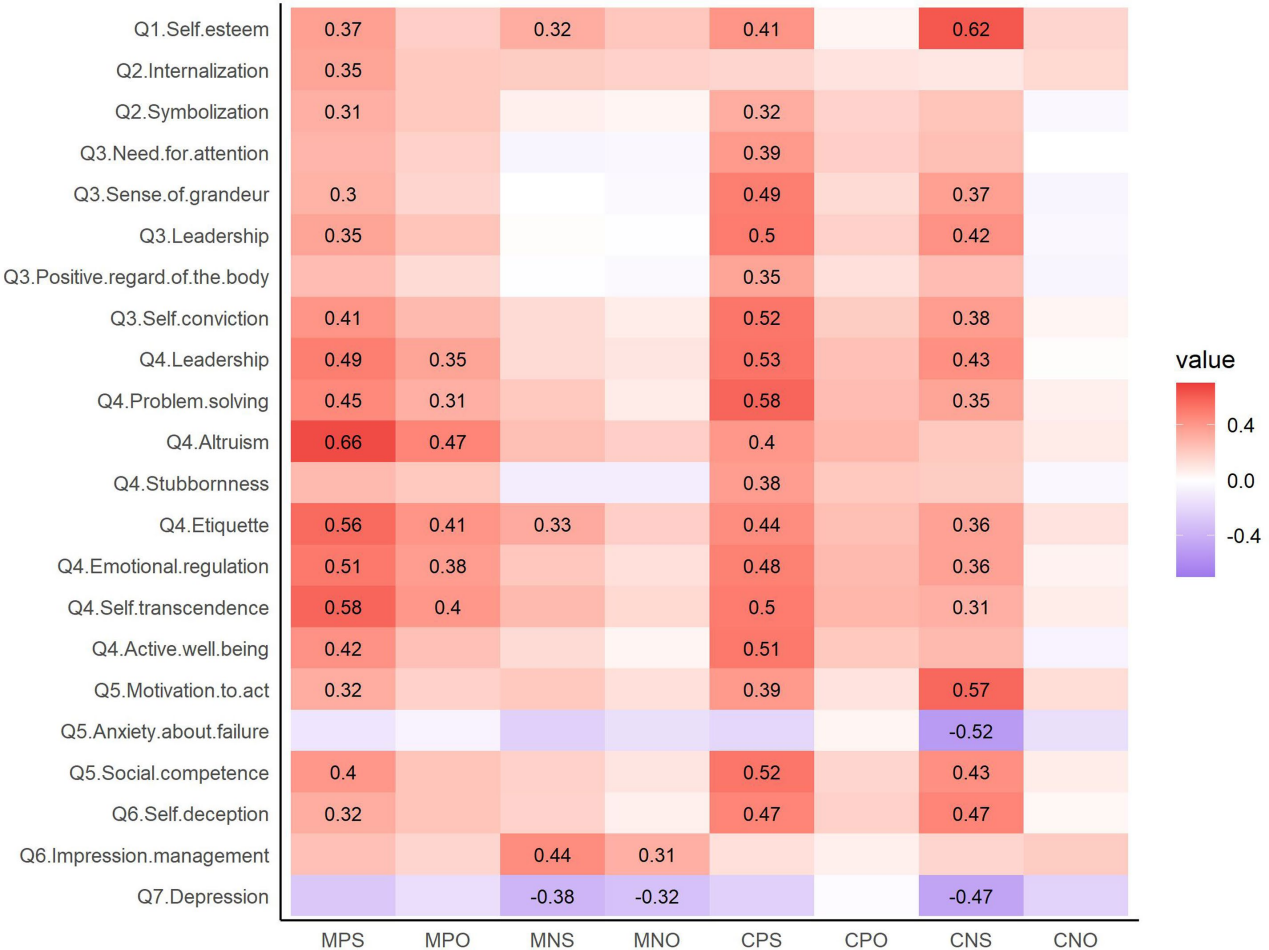
Heatmap of the correlation coefficients between self-and average other-evaluation and psychobehavioral characteristics. S: self-evaluation; O: average other-evaluation. Other abbreviations are the same as in Figure 4.

The psychobehavioral characteristics were correlated with self-and other-evaluations under the two moral conditions (Figure 5). Self-and other-evaluations under the positive moral condition were related to all factors on the Power to Live scale except stubbornness and active well-being, while self-and other-evaluation under the negative moral condition were related to impression management on the BIDR and depression. In addition, some characteristics were related to self-evaluation but not to other-evaluation. Self-evaluation under the positive moral condition was related to 10 characteristics, including self-esteem, internalization, and symbolization on the MI; sense of grandeur, leadership, and self-conviction on the NPI; active well-being on the Power to Live Scale; motivation to act and social competence on the GSE; and self-deception on the BIDR. Self-evaluation under the negative moral condition was associated with self-esteem and etiquette on the Power to Live Scale.

Only self-evaluation was related to psychobehavioral characteristics under the two competence conditions. Self-evaluation under both conditions was correlated with 12 psychobehavioral characteristics, including self-esteem, sense of grandeur, leadership, and self-conviction on the NPI; leadership, problem-solving, etiquette, emotional regulation, and self-transcendence on the Power to Live Scale; motivation to act and social competence on the GSE; and self-deception on the BIDR scale. Additionally, self-evaluation under the positive competence condition was related to six characteristics, including symbolization on the MI; need for attention and positive regard of the body on the NPI; and altruism, stubbornness, and active well-being on the Power to Live Scale. Self-evaluation under the negative competence condition was related to anxiety about failure on the GSE and depression.

## Discussion

In this study, we aimed to deepen our understanding of the psychological mechanisms of the BTAE by investigating the BTAE in four domains and examining the relationships between psychobehavioral characteristics and the BTAE, as well as the relationship between psychobehavioral characteristics and self−/other-evaluations. First, we found that the BTAE existed only under the negative moral condition, whereas the WTAE was found under both the positive and negative competence conditions. Second, we verified the uniquely prevalent illusion, where the BTAE was not correlated with any of the psychobehavioral characteristics in the negative moral domain but was related to them under the three other conditions. Third, self-and other-evaluations in the moral domain had similar correlations with psychobehavioral characteristics, whereas only self-evaluation was correlated with the psychobehavioral characteristics in the competence domain. These results reconcile two opposing views regarding the BTAE–personality relationship (i.e., self-centrality breeds self-enhancement vs. the uniquely prevalent illusion).

We considered two independent psychological mechanisms potentially underlying these complex findings. First, there appears to be a process that explains why the BTAE can often be found in the moral domain under self-protective motives. Second, there appears to be a process that complicates the expression of the BTAE in the moral domain by biasing not only self-evaluation but also evaluation of others.

The presence of BTAE in the moral domain and WTAE in the competence domain is consistent with previous studies conducted in Japanese cultures. Several studies have observed the BTAE in the moral domain and WTAE in the competence domain when examining their stimuli in detail (Brown and Kobayashi, 2002; Sedikides et al., 2003; Hamamura et al., 2007). For instance, Japanese participants rated themselves as better than most other students on moral adjectives (e.g., being friendly, responsible, valuing friends, and being well-liked) but worse on “competence.” Similar results have been reported in other studies. Researchers found WTAE when using a mix of competence-related adjectives (half of stimuli) and those from other domains, such as attractiveness, honesty, getting along well with others, cooperativeness, and happiness (Endo et al., 2000).

Social acceptance may explain the presence and large degree of the BTAE in the moral domain, particularly in the context of self-protective motives. People have a fundamental need to belong and a strong desire to connect with others (Baumeister and Leary, 1995). Consequently, information threatening social acceptance triggers a powerful defense mechanism. According to social perception theory, morality is a key social perception dimension that determines whether people are accepted, as good or bad intentions can significantly impact others’ interests (Fiske et al., 2007; Abele et al., 2021). Unethical individuals are perceived as more threatening and are more likely to be rejected (van der Lee et al., 2017). People instinctively avoid associating with unethical information, rejecting objects with immoral attributes (Kupfer and Giner-Sorolla, 2021). When participants evaluated their immoral traits, the risk of decreased social acceptance was high. Consequently, they had a strong incentive to exhibit the BTAE regarding immoral traits to mitigate threats to their social acceptance.

The occurrence of the WTAE in the competence domain may also be attributed to considerations of social acceptance. The WTAE is often interpreted as a form of modesty regarding one’s abilities and is perceived as a strategy to avoid offending others. In contrast, presenting oneself as superior can be perceived as challenging or threatening to others (Takata, 1987; Allison et al., 1989; Heine et al., 2000; Suzuki and Yamagishi, 2004; Yamagishi et al., 2012). This effect can even be observed in anonymous situations (Suzuki and Yamagishi, 2004; Yamagishi et al., 2012). People may exhibit the WTAE as a way to maintain social acceptance and avoid potential social conflicts.

Our findings, which show a lack of correlation between psychobehavioral characteristics and BTAE in the moral domain—particularly regarding negative morality—support the notion of a uniquely prevalent illusion rather than the “self-centrality breeds self-enhancement” principle. Consistent with the unique prevalent illusion (Tappin and McKay, 2017), we observed that the absence of a correlation between BTAE in the moral domain and psychobehavioral characteristics is particularly evident in the negative moral domain. This lack of correlation may be attributed to the similar patterns of self-and other-evaluations related to personality traits. Consequently, the BTAE-personality association might be diminished or obscured when using indirect methods to assess BTAE.

We speculate that the similar perceptual bias toward self-and other-evaluation in the moral domain is related to mutual trust, which has evolved under the pressures of social survival. In the positive moral condition, participants with higher scores for survival-oriented traits evaluated both themselves and others more positively. From an evolutionary perspective, human survival often depends on collaboration to secure resources and compete with other groups (Tomasello et al., 2012). This cooperation necessitates mutual trust and the demonstration of trustworthiness. Consequently, people tend to view themselves and others as trustworthy (Fiske et al., 2007; Tomasello and Vaish, 2013). The Power to Live scale, which measures factors associated with disaster survival, was developed following a real earthquake and its aftermath. In both disaster and everyday situations, cooperation with others is essential for mutual benefit, leading people to believe in the inherent moral goodness of others to enhance their chances of survival.

In the negative moral condition, we observed a positive correlation between self−/other-evaluation and impression management, as well as a negative correlation with depression. Impression management involves the tendency to adjust one’s behavior to create a favorable impression and gain others’ favor (Paulhus and John, 1998). Criticizing others, or “speaking ill of,” can damage one’s own moral reputation and lead to disapproval. Therefore, to maintain a positive image, individuals may rate both themselves and others as less immoral. Additionally, depression—a state characterized by persistent negative mood—is associated with increased risk of suicide and self-harm (Hawton et al., 2012). Negative self-and other-evaluations are known to contribute to depression (Tabachnik et al., 1983). Our findings suggest that negative perceptions of self and others in terms of immorality may adversely affect mental health.

We propose a framework consisting of social perception dimension and motivation to investigate BTAE. In this study, the BTAE was seen only in the context of morality and the self-protection motive. These findings indicate the importance of the interplay between the social perception dimension and motivation. At the average level, the presence of the BTAE and the WTAE may be influenced by social acceptance. Moreover, the limited number of correlations between the BTAE in moral domains and psychobehavioral characteristics may be attributed to similar perceptual biases between self-and other-evaluations. This may potentially be driven by the mutual trust conducive for social survival. These findings could aid investigations of the BTAE by showing the importance of considering social perception dimension and motivation. They also shed light on two underlying mechanisms of the BTAE, which may stem from mutual trust. In particular, we highlight the intricate processes of biased person perception involved in BTAE. These processes provide a nuanced and advanced comprehension of BTAE that extends beyond classical assumptions. In summary, this study provides insights into the sociocultural dynamics of the BTAE, the relationships between psychobehavioral characteristics and perceptions, and the roles of self-and other-perception in mutual trust.

### Limitation and future research

This study had several limitations. First, we focused only on morality and competence; we did not consider sociability, which is another important social perception dimension (Goodwin et al., 2014). Future studies should explore sociability in conjunction with morality and competence to better understand the distinct impacts of each social perception dimension on the BTAE. Second, our participants were predominantly from a younger generation, which may limit the generalizability of our findings to other age groups. Future studies should aim to include participants from a wider age range to enhance the applicability of the results. Third, we did not randomize the order of self-and other-evaluations, with participants completing before other-evaluation. Future research should address the potential influence of task order on results. Additionally, our survey comprised 310 items, which may have contributed to participant fatigue. Although 79% of participants completed the survey within 30 min, the extensive length could have affected attention. Future studies should consider reducing the number of items or incorporating breaks to mitigate fatigue and maintain participant engagement.

Our work highlights the need of considering both social perception dimension and motivation factors in investigating the BTAE, as well as the relationships between psychobehavioral characteristics and BTAE. Future research should examine the effects of social acceptance and rejection on BTAE across different domains. For instance, it would be valuable to explore whether social acceptance and rejection influence different tendencies within BTAE or WTAE. Investigating the intervention effects of social acceptance and rejection on BTAE, as well as individual differences in perceptions of acceptance and rejection, could provide further insights. Additionally, given the increasing interest in the impact of demographic factors such as age, sex, and culture on BTAE, future studies should incorporate demographic information and culturally specific social perception dimensions. This would offer a deeper understanding of how BTAE varies across different populations (e.g., generations and sexes) and cultural contexts. Moreover, future research could use fMRI to investigate neural processing differences between self-evaluation and other-evaluation, as well as their relationship with psychobehavioral characteristics. This approach would shed light on the neural mechanisms underlying these evaluations and help identify the cognitive processes associated with self-and other-evaluations.

## Conclusion

In this study, the BTAE occurred in the moral domain under the self-protection motive, and the WTAE occurred in the competence domain under the self-enhancement and self-protection motives. The BTAE in the moral domain was associated with few psychobehavioral characteristics, and in particular showed no correlation with the negative moral condition. In contrast, the BTAE in the competence domain was related to many characteristics. Moreover, similar patterns of correlations between psychobehavioral characteristics and self-and other-perceptions were observed in two moral domains, which may explain the small number of associations between BTAE and characteristics in the two moral domains. Psychobehavioral characteristics were exclusively linked to self-evaluation in two competence domains. This study implies that two psychological mechanisms underlie the BTAE. First, social acceptance may explain the presence of the BTAE and the WTAE. Second, the limited associations between the BTAE in the moral domain and psychobehavioral characteristics may be due to similarly biased self-and other-perceptions at the individual level, potentially driven by evolutionary pressure for mutual trust. This study elucidates the sociocultural dynamics of the BTAE in the context of social perception dimensions and motivation. It provides a framework for future studies to assess the BTAE further and contributes to our understanding of the associations among the BTAE, personal perceptions, and social interactions.

## Data Availability

The datasets presented in this study can be found in online repositories. The names of the repository/repositories and accession number(s) can be found at: https://osf.io/xm5hw/.
