# Supplementary material for: The psychological mechanisms of the better-than-average effect in the moral and competence domains under self-enhancement and self-protection motives among young Japanese adults
Source: Front Psychol. 2024 Oct 22;15:1367568. doi: 10.3389/fpsyg.2024.1367568 (PMC11534810; doi:10.3389/fpsyg.2024.1367568)
Supplement: Supplementary file 1 [file Data_Sheet_1.pdf]

Supplementary Material

The psychological mechanisms of the better-than-average effect in the moral and competence domains under self-enhancement and self-protection motives among young Japanese adults

Yi Ding 1,2,3* and Motoaki Sugiura 1,4

1. Institute of Development, Aging and Cancer, Tohoku University, Sendai, Japan
2. Graduate School of Medicine, Tohoku University, Sendai, Japan
3. Japan Society for the Promotion of Science, Tokyo, Japan
4. International Research Institute of Disaster Science, Tohoku University, Sendai, Japan

*** Correspondence:**
Yi Ding
dingyi9508@outlook.com

Method

Participants

Poor data quality is an unavoidable problem in online surveys (Chmielewski & Kucker, 2020). Low-quality data can influence the associations between variables. It has been suggested that participants with very fast response times and inconsistent responses should be excluded from analyses (Wood et al., 2017). In the present study, we used cut-offs based on response time and response consistency to exclude certain participants from the analysis. Response consistency was sensitively reflected in the scales containing reverse items.

We calculated the moving average of the reliability coefficients for the self-esteem, self-efficacy, and social desirability scales, which all included reverse items (Sakano & Mitsuhiko, 1986; Tani, 2008; Yamamoto et al., 1982). With a subset size of 100 participants, 901 subsets of data were obtained (e.g., [1:99]; [2:100], etc.) according to response time (from fast to slow). We calculated internal consistency Cronbach's alpha scores to determine the response consistency of the groups. The reliability coefficients were high for all three scales when the reaction time was more than 8 minutes (Supplementary figure 1).

Results

Supplementary figure 2 illustrates the mean BTAE scores in the four domains, using data that do not exclude satisfiers based on reaction time. The main results that negative moral BTAE existed didn't change. Specifically, the one-sample *t*-test indicated that the BTAE score for the positive moral condition did not differ significantly from 0 [$t(999) = 1.538$, $p = 0.124$, Cohen's $d = 0.049$, 95% CI: –0.013, 0.111]. The BTAE score for the negative moral condition was greater than 0, indicating that people considered themselves less immoral than others [$t(999) = 10.407$, $p < 0.001$, Cohen's $d = 0.329$, 95% CI: 0.265, 0.393]. The BTAE scores for the positive and negative competencies were less than 0, indicating that the participants

considered themselves less capable in the positive competency domain [$t(999) = -4.872$, $p < 0.001$, Cohen's $d = 0.154$, 95% CI: 0.092, 0.216] and more incompetent than others [$t(999) = -4. 829$, $p < 0.001$, Cohen's $d = 0.201$, 95% CI: 0.090, 0.215].

Supplementary figure 1

The moving average Cronbach's α for the self-efficacy, self-esteem, and social desirability scales

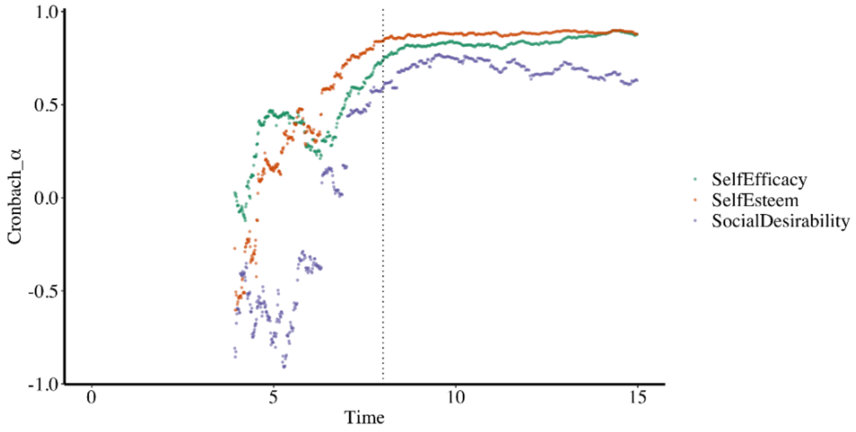

Note. The dashed line indicates a response time of 8 minutes.

Supplementary figure 2

Raincloud plot. (A) The mean BTAE scores and (B) distribution of raw BTAE scores in the four conditions

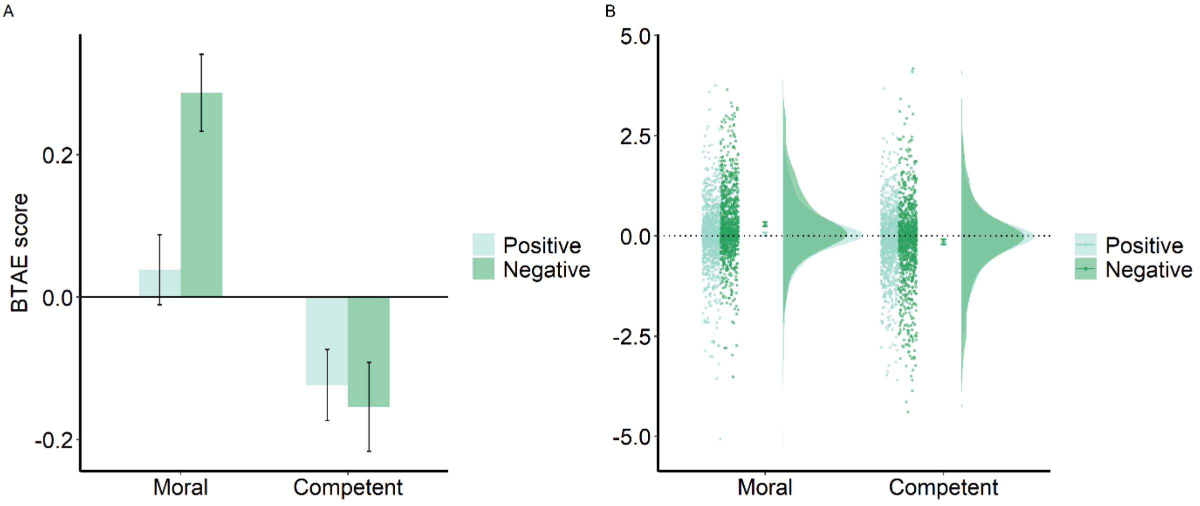

Note. Error bars are the 95% CIs.

Supplementary table 1 represents the results of correlation analysis in detail, using data that do not exclude satisfiers based on reaction time. The main finding of an independent association of negative moral BTAE with psychobehavioral characteristics didn't change (the largest coefficient was for etiquette; $r = 0.183$). And we found a lacking significant association between "positive moral BTAE", and psychobehavioral characteristics reached an $|r|$ of 0.3 (the largest coefficient was for altruism; $r = 0.265$).

Supplementary table 1

Person correlation results between four types of the BTAE and psychobehavioral characteristics

	MP	MN	CP	CN
**Power to live**				
Leadership	0.183	0.043	0.292	**0.392**
Problem-solving	0.182	0.177	**0.301**	0.254
Altruism	0.265	0.102	0.140	0.146
Stubbornness	0.101	0.029	0.186	0.201
Etiquette	0.209	0.183	0.174	0.214
Emotional regulation	0.181	0.117	0.213	0.280
Self-transcendence	0.234	0.157	0.213	0.219
Active well-being	0.207	0.121	0.266	0.271
**Self-efficacy**				
Motivation to act	0.171	0.094	0.287	**0.421**
Anxiety about failure	-0.067	-0.079	-0.197	**-0.335**
Social competence	0.194	0.076	**0.331**	**0.303**
**Self-esteem**	0.217	0.100	**0.357**	**0.450**
**Moral identity**				
Internalization	0.171	0.073	0.048	-0.037
Symbolization	0.117	0.023	0.165	0.246
**Narcissism**				
Need for attention	0.125	-0.053	0.224	0.232
Sense of grandeur	0.135	-0.021	**0.317**	**0.367**
Leadership	0.142	-0.021	**0.315**	**0.400**
Positive regard of the body	0.120	-0.022	0.239	0.286
Self-conviction	0.176	0.055	**0.327**	**0.305**
**Depression**	-0.150	-0.109	-0.201	-0.228
**Social desirability**				
Self-deception	0.116	0.104	0.294	**0.406**
Impression management	0.132	0.155	0.085	-0.003

Note. $|r| > 0.3$ are in bold. MP: positive morality; MN: negative morality; CP: positive competence; CN: negative competence.

Overview of Stimuli Survey

Stimuli (adjectives) were selected based on two surveys' results. Participants were recruited via Lancers, an online Japanese recruitment agency, and surveys were created using online survey software (Qualtrics, USA). In total, 592 personality trait-related adjectives were categorized into four domains (positive morality, negative morality, positive competence, negative competence). We included 40 adjectives in each domain (160 adjectives in total). We then assessed the 160 adjectives in terms of social value, valence, and familiarity. Ultimately, 20 were included in each domain as stimuli (80 adjectives in total).

Personality Trait-related Adjective Categorization Survey

Participants

We recruited 123 participants; 12 satisficers were excluded because they failed the attention test (i.e., failed to correctly distinguish moral- and competence-related words). We obtained 119 valid data sets from 88 males (mean age = 39.40 ± 7.60 years).

Procedure

To obtain 160 personality trait-related adjectives, we created a list of 592 Japanese adjectives from two corpora (Aoki, 1971; Murakami, 2002). Participants were instructed to choose the most suitable domain for each of the 592 adjectives, from among the four domains of positive moral, negative moral, positive competent, and negative competent. If they were unsure as to which domain the adjective belonged, or did not understand its meaning, they could respond "*Do not know*" or *"Do not understand"*, respectively.

Results

We excluded adjectives with a "*Don't know*" response from at least three participants. We included only adjectives 3–6 characters in length. We then selected the 40 adjectives chosen most frequently for a given domain, and least frequently for the other domains (160 adjectives in total).

Personality Trait Adjective-related Selection Survey

Participants

We recruited 84 participants and ultimately included data from 52 of them (27 males; mean age = 26.5 ± 2.35 years). In this survey, 18 satisficers were excluded because they failed the attention task (i.e., failed to correctly distinguish moral- and competence-related words), and 14 participants aged above 30 years were excluded to avoid age effects associated with the degree of familiarity with the word list.

Procedure

Participants were asked to rate the 160 adjectives according to three properties: social value from 1 (*very moral*) to 7 (*very competent*), valence from 1 (*very negative*) to 7 (*very positive*), and familiarity from 1 (*not familiar at all*) to 7 (*very familiar*).

Results

We selected 20 adjectives for each domain (80 adjectives in total) from among the 160 adjectives based on familiarity and representativeness. We excluded unfamiliar (i.e., familiarity score < 4) adjectives. We then selected adjectives that had higher scores for social value and valence in the corresponding domains. Supplementary table 2 presents the Japanese word list and English translations.

Supplementary table 2

The Japanese word list and English translations for each domain

MP	MN	CP	CN
人情に厚い<br>warmhearted	薄情な<br>heartless	かしこい<br>smart	とろい<br>stupid
善意のある<br>well-meaning	憎らしい<br>hateful	着実な<br>steady	忘れっぽい<br>forgetful
人のよい<br>kindhearted	嫌らしい<br>horrible	力強い<br>powerful	忍耐力のない<br>impatient
親身な<br>kind	意地悪な<br>mean	すきがない<br>hardworking	にぶい<br>dull
同情心のある<br>sympathetic	人をけなす<br>critical	綿密な<br>cautious	中途半端な<br>halfway
礼儀正しい<br>polite	高圧的な<br>overbearing	ぬけ目のない<br>shrewd	か弱い<br>weak
素直な<br>honest	うさんくさい<br>suspicious	注意深い<br>careful	ぼそぼそ話す<br>slow
友情のある<br>friendly	冷酷な<br>cruel	活動的な<br>active	危なっかしい<br>unreliable
飾りのない<br>simple	厚かましい<br>shameless	説得力のある<br>convincing	軟弱な<br>cowardly
おだやかな<br>mild	中傷する<br>slanderous	念入りな<br>conscientious	自信のない<br>unconfident
親切な<br>cordial	浮気っぽい<br>flirtable	すばやい<br>alert	実行力のない<br>inactive
親心のある<br>affectionate	身勝手な<br>selfish	努力する<br>diligent	どんくさい<br>clumsy
情け深い<br>compassionate	ひがむ<br>perverse	徹底する<br>thorough	口下手な<br>inarticulate
人間味のある<br>humane	憎たらしい<br>damnable	つよい<br>strong	よわい<br>fragile
優しい<br>gentle	八つ当たりする<br>irritable	頼もしい<br>reliable	探求心のない<br>uninquisitive
温かい<br>warm	ふてぶてしい<br>brazen	つきつめる<br>absorbed	頼り無い<br>undependable
正直な<br>virtuous	嫌みたらしい<br>sarcastic	細心な<br>conscientious	弱々しい<br>feeble
義理堅い<br>dutiful	告げ口をする<br>telltale	入念な<br>scrupulous	そそっかしい<br>impetuous
謙虚な<br>modest	勝手な<br>selfish	聞き上手な<br>attentive	不安定な<br>unstable
円満な<br>peaceful	意地汚い<br>Greedy	几帳面な<br>well-organized	弱腰な<br>weak-kneed

Reference

Aoki, T. (1971). A psycho-lexial study of personality trait words selection, classification and desirability ratings of 455 words. *Jpn. J. Psychol*, *42*, 1–13.

Chmielewski, M., & Kucker, S. C. (2020). An MTurk Crisis? Shifts in Data Quality and the Impact on Study Results. *Social Psychological and Personality Science*, *11*(4), 464–473. https://doi.org/10.1177/1948550619875149

Murakami, Y. (2002). A collection of basic personality trait words. *The Japanese Journal of Personality*, *11*(1), 35–49. https://doi.org/10.2132/jjpjspp.11.1_35

Sakano, Yuji., & Mitsuhiko, T. (1986). The general self-efficacy scale (GSES): Scale development and validation. *Japanese Journal of Behavior Therapy*, *12*(1), 73–82.

Tani, I. (2008). Development of Japanese Version of Balanced Inventory of Desirable Responding (BIDR-J). *The Japanese Journal of Personality*, *17*(1), 18–28. https://doi.org/10.2132/personality.17.18

Wood, D., Harms, P. D., Lowman, G. H., & DeSimone, J. A. (2017). Response Speed and Response Consistency as Mutually Validating Indicators of Data Quality in Online Samples. *Social Psychological and Personality Science*, *8*(4), 454–464. https://doi.org/10.1177/1948550617703168

Yamamoto, M., Matsui, Y., & Yamanari, Y. (1982). The structure of perceived aspects of self. *The Japanese Journal of Educational Psychology*, *30*(1), 64–68. https://doi.org/10.5926/jjep1953.30.1_64
